# Fertility and Regional Post-Insemination Cytokine Responses in Hens Following Use of Avian β-Defensin 1 or Kanamycin in Rooster Semen Extenders

**DOI:** 10.3390/ani16213525

**Published:** 2026-11-07

**Authors:** Ruthaiporn Ratchamak, Eakapol Wangkahart, Supakorn Authaida, Wuttigrai Boonkum, Vibuntita Chankitisakul

**Affiliations:** 1 Division of Animal Science, Department of Agricultural Technology, Faculty of Technology, Mahasarakham University, Maha Sarakham 44150, Thailand; ruthaiporn.r@msu.ac.th; 2 The Research and Development Network Center of Animal Breeding and Omics, Khon Kaen University, Khon Kaen 40002, Thailand; wuttbo@kku.ac.th; 3 Division of Fisheries, Department of Agricultural Technology, Faculty of Technology, Mahasarakham University, Maha Sarakham 44150, Thailand; eakapol.w@msu.ac.th; 4 Laboratory of Fish Immunology and Nutrigenomics, Applied Animal and Aquatic Sciences Research Unit, Division of Fisheries, Faculty of Technology, Mahasarakham University, Maha Sarakham 44150, Thailand; 5 Department of Animal Science, Faculty of Agriculture, Khon Kaen University, Khon Kaen 40002, Thailand; supakorn.u@kkumail.com

**Keywords:** bacterial contamination, antimicrobial peptide, gene expression, inflammatory response, artificial insemination

## Abstract

Successful artificial insemination in poultry depends on both sperm functional competence and an appropriate immune environment within the female reproductive tract. This study investigated whether supplementing rooster semen extenders with kanamycin or avian β-defensin 1 (AvBD1) affected reproductive performance and regional post-insemination cytokine responses. Semen from 30 Thai native roosters was pooled and diluted in IGGKPh extender without antimicrobial supplementation (Control) or supplemented with kanamycin (31.2 µg/mL; KAN) or AvBD1 (7.5 µg/mL). Thirty-two Thai native hens were assigned to the three semen-inseminated treatments or a sham-inseminated NaCl group. Fertility and hatchability of fertile eggs were evaluated over 10 weeks. At 24 h after the final insemination, four hens per treatment were sampled for analysis of TNF-α, IL-1β, IL-6, IL-8, and IL-10 mRNA expression in the vagina, uterovaginal junction (UVJ), and infundibulum. Fertility was higher in the KAN and AvBD1 groups than in the Control group (70.23% and 72.81% vs. 65.27%; *p* < 0.05), whereas no significant difference was detected in hatchability of fertile eggs. No significant treatment effects on the measured cytokines were detected in the vagina. Treatment-associated differences in selected cytokines were observed in the UVJ and infundibulum, with most significant pairwise differences involving the NaCl sham group. AvBD1 showed lower IL-8 expression in the infundibulum than the antimicrobial-free Control. No significant difference in fertility was detected between KAN and AvBD1; however, the study was not designed to establish equivalence or non-inferiority between these treatments. These findings support further evaluation of AvBD1 as a candidate antibiotic-sparing component of poultry semen extenders.

## Simple Summary

Antibiotics are commonly added to rooster semen extenders to control bacterial contamination, but concerns about antimicrobial resistance have increased interest in antibiotic-sparing approaches. This study examined reproductive outcomes and post-insemination cytokine responses associated with supplementing rooster semen extenders with kanamycin (KAN) or avian β-defensin 1 (AvBD1). Thai native chicken hens were artificially inseminated weekly for 10 weeks with semen diluted in extender without antimicrobial supplementation, with KAN, or with AvBD1. Fertility was higher in both supplemented groups than in the antimicrobial-free Control, whereas hatchability of fertile eggs did not differ significantly. Treatment-associated cytokine expression patterns were observed in selected reproductive regions. Because bacterial load and direct antimicrobial activity were not measured at insemination, the mechanisms underlying these findings remain uncertain and may involve effects on sperm, microbial exposure, or interactions with the female reproductive tract. These findings support further evaluation of AvBD1 as a potential antibiotic-sparing component of rooster semen extenders.

## 1. Introduction

Successful artificial insemination (AI) in poultry depends on maintaining sperm functional competence during semen collection, preparation, and insemination. Bacterial contamination can occur during semen collection and handling and may adversely affect sperm function, supporting the use of antimicrobial agents in poultry semen extenders [1,2]. However, routine use of conventional antibiotics has raised concerns regarding antimicrobial resistance and has encouraged the development of alternative antimicrobial strategies [3,4].

Antimicrobial peptides, particularly avian β-defensins, are promising candidates because of their broad antimicrobial activity and endogenous roles in avian host defense [5,6]. In our previous study, AvBD1 supplementation reduced viable bacterial load and oxidative damage and maintained sperm quality during chilled storage, while fertility was higher than that obtained with an antimicrobial-free extender [7]. That study primarily addressed semen preservation before insemination. In contrast, the present study used freshly prepared semen and focused on responses within the recipient hen by evaluating reproductive outcomes together with post-insemination cytokine expression in anatomically distinct regions of the reproductive tract. Whether insemination with AvBD1- or kanamycin-supplemented semen is associated with regional cytokine responses within the female reproductive tract remains unclear.

After insemination, sperm encounter distinct mucosal environments during transport, selection, storage, and fertilization, while the reproductive tract defends against microorganisms [8]. The vagina is directly exposed to the inseminated formulation and participates in sperm selection and clearance, whereas the uterovaginal junction (UVJ) contains specialized sperm storage tubules (SSTs) that support prolonged sperm survival [8,9,10]. The infundibulum receives sperm released from storage and is the site of fertilization. Experimental evidence showing localized transcriptional responses following insemination further indicates that immune responses are not uniform throughout the reproductive tract [11,12,13]. These functional and immunological differences provide a biological basis for evaluating the three regions separately.

Cytokines provide useful molecular indicators of the post-insemination response. TNF-α and IL-1β are early pro-inflammatory mediators involved in epithelial activation and inflammatory signaling, whereas IL-6 participates in inflammatory regulation and epithelial homeostasis, and IL-8 promotes recruitment of heterophils and other leukocytes to mucosal tissues [13,14,15,16]. Excessive or prolonged local inflammatory responses may contribute to sperm degradation and clearance, whereas a less inflammatory environment within the UVJ may favor sperm survival in the SSTs [8,13]. In contrast, IL-10 is a key counter-regulatory cytokine that limits excessive inflammation and supports immune homeostasis [17]. Accordingly, these five cytokines were selected to represent complementary aspects of inflammatory initiation, leukocyte recruitment, and counter-regulatory signaling relevant to the local environment encountered by sperm during transport, storage, and fertilization. Their expression was used as an indicator of local transcriptional responses rather than as direct evidence of functional effects on sperm or reproductive tissues.

Therefore, the present study evaluated the effects of supplementing rooster semen extenders with kanamycin or AvBD1 on fertility and regional cytokine gene expression in the vagina, UVJ, and infundibulum of laying hens following AI. We hypothesized that antimicrobial supplementation would increase fertility relative to an antimicrobial-free extender and would be associated with treatment-dependent cytokine expression patterns within the female reproductive tract. Because the UVJ contains the SSTs and represents the principal site of prolonged sperm storage, it was considered a region of particular biological interest; however, no specific directional response was prespecified for individual cytokines.

## 2. Materials and Methods

### 2.1. Ethical Approval

All experimental procedures involving animals were conducted in accordance with institutional guidelines for the care and use of animals in research. The experimental protocol was reviewed and approved by the Institutional Animal Care and Use Committee of Khon Kaen University, Thailand (Approval No. IACUC-KKU-28/69).

### 2.2. Animals and Husbandry

Thirty sexually mature Pradu Hang Dum roosters, 16 months of age, were used as semen donors. The roosters were housed individually in cages measuring 60 × 45 × 45 cm under a 16 h light/8 h dark photoperiod. Each rooster received 130 g/day of feed, with water provided ad libitum.

Thirty-two Kai Mook Esan hens, 8 months of age and with a laying rate greater than 80%, were used as recipients for artificial insemination. The hens were housed individually under the same environmental and lighting conditions as the roosters. Each hen received 110 g/day of feed, with water provided ad libitum throughout the experimental period.

Both roosters and hens received the same commercial breeder diet (Balance 924; Betagro Company Limited, Nakhon Ratchasima, Thailand), containing 17% protein, 3% fat, 6% fiber, and 13% moisture according to the manufacturer’s specification.

### 2.3. Semen Extender Preparation

Kanamycin sulfate derived from Streptomyces kanamyceticus was purchased from Sigma-Aldrich (St. Louis, MO, USA; product no. K400). AvBD1 was purchased from Beijing Tsingke Biotech Co., Ltd. (Beijing, China; product no. JT-211645). Unless otherwise specified, all other chemicals were of analytical grade and purchased from Sigma-Aldrich.

The IGGKPh semen extender was prepared according to Ratchamak et al. [18]. Briefly, 0.14 g potassium citrate, 1.40 g sodium glutamate, 0.21 g sodium dihydrogen phosphate, 0.98 g disodium hydrogen phosphate, 0.90 g glucose, and 0.90 g inositol were dissolved in deionized water to a final volume of 100 mL. The extender was filter-sterilized through a 0.22 µm syringe filter and stored at 4 °C until use.

### 2.4. Experimental Design

**Figure 1:**
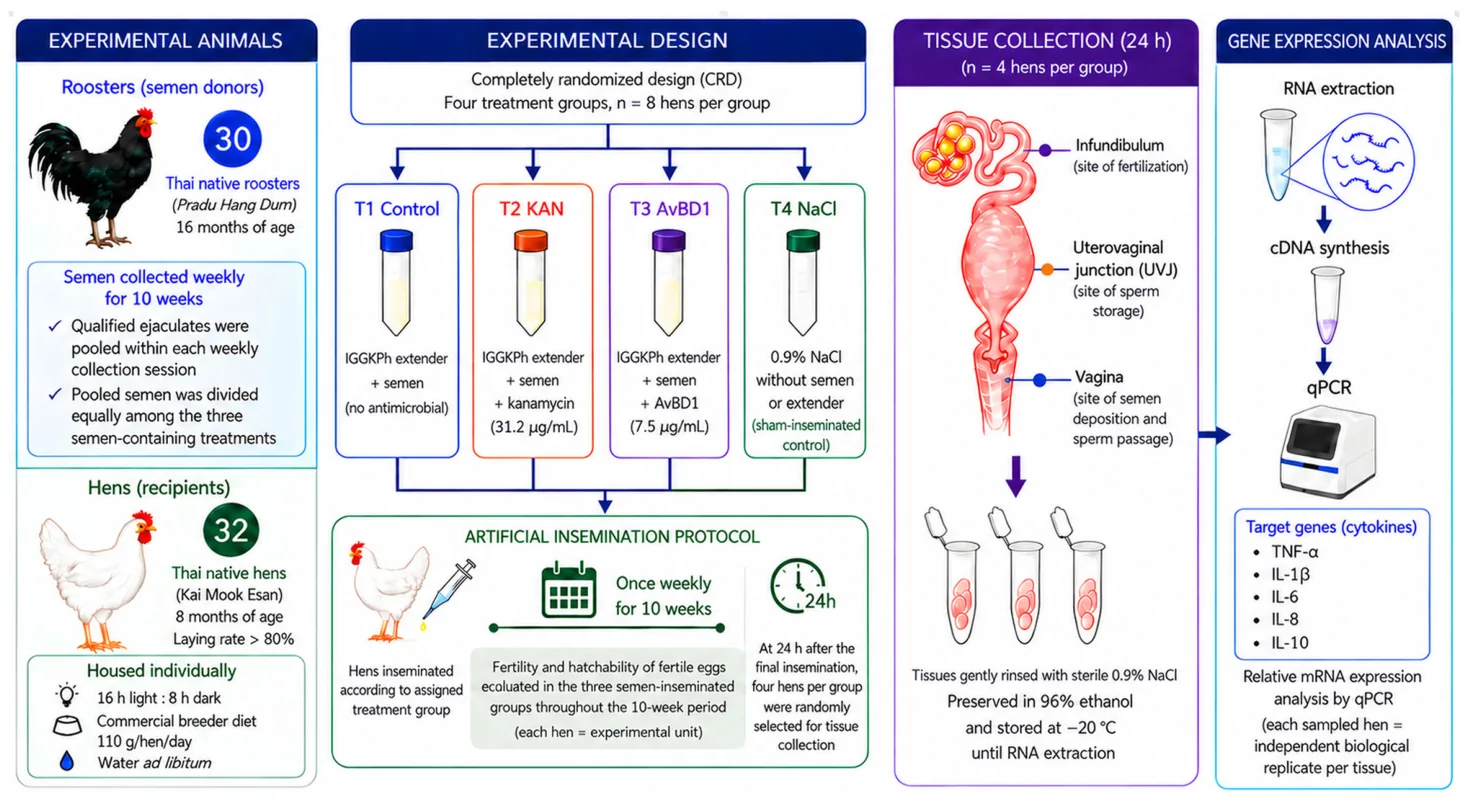
Experimental design of the artificial insemination and tissue-collection trial. Semen collected from 30 Pradu Hang Dum roosters was pooled and divided among three treatments: semen diluted with IGGKPh extender without antimicrobial supplementation (Control), semen diluted with IGGKPh extender containing kanamycin at 31.2 µg/mL (KAN), and semen diluted with IGGKPh extender containing avian β-defensin 1 (AvBD1) at 7.5 µg/mL (AvBD1). A fourth group received 0.9% NaCl without semen or extender and served as the sham-inseminated control (NaCl). Thirty-two Kai Mook Esan hens were randomly allocated to the four treatments (n = 8 hens per treatment). Artificial insemination was performed weekly for 10 weeks, and fertility and hatchability of fertile eggs were evaluated in the three semen-inseminated groups. At 24 h after the final insemination, four hens from each treatment were randomly selected for collection of the vagina, uterovaginal junction, and infundibulum. Relative mRNA expression of TNF-α, IL-1β, IL-6, IL-8, and IL-10 was subsequently determined by quantitative real-time PCR. Each sampled hen represented one independent biological replicate within each reproductive tissue.

A completely randomized design was used to evaluate the effects of antimicrobial supplementation of rooster semen extenders on fertility and regional cytokine gene expression in the hen reproductive tract following artificial insemination (Figure 1). Thirty-two Kai Mook Esan hens were randomly allocated by drawing lots to four treatment groups (n = 8 hens per treatment): (1) semen diluted with IGGKPh extender without antimicrobial supplementation (Control); (2) semen diluted with IGGKPh extender containing kanamycin at 31.2 µg/mL (KAN); (3) semen diluted with IGGKPh extender containing AvBD1 at 7.5 µg/mL (AvBD1); and (4) sham insemination with 0.9% NaCl without semen or extender (NaCl).

The NaCl group served as a procedural sham control and received 0.1 mL of sterile 0.9% NaCl using the same insemination procedure as the semen-inseminated groups. Because this group contained neither semen nor IGGKPh extender, it was not an extender-matched control.

The kanamycin and AvBD1 concentrations refer to their concentrations in the prepared IGGKPh extender before mixing with semen. Kanamycin at 31.2 µg/mL was selected based on previous studies using this concentration in rooster semen extenders [19], whereas AvBD1 at 7.5 µg/mL was selected based on previous evidence demonstrating antimicrobial activity against poultry-associated bacteria without detectable cytotoxicity toward chicken HD11 cells within the tested concentration range [20]. Both concentrations were subsequently evaluated in IGGKPh extender for rooster semen preservation in our previous study [7]. Following dilution at a ratio of 1:3 (*v*/*v*; semen), the corresponding final concentrations in the insemination mixture were 23.4 µg/mL kanamycin and 5.625 µg/mL AvBD1, respectively. The two compounds were therefore evaluated at previously supported working concentrations rather than at equal mass, equimolar, or equivalent antimicrobial-potency doses.

Semen was collected from 30 Pradu Hang Dum roosters. Ejaculates meeting the predefined quality criteria were pooled to reduce variation among individual semen donors and then divided into equal aliquots for the Control, KAN, and AvBD1 treatments. Hens in the NaCl group received 0.9% NaCl using the same insemination procedure.

Fertility and hatchability of fertile eggs were evaluated in the three semen-inseminated groups over the 10-week experimental period. Each hen was considered one experimental unit for reproductive performance. At 24 h after the final insemination, four hens from each treatment group were randomly selected for tissue collection using the same lottery method. Each sampled hen represented one independent biological replicate for gene expression analysis within each reproductive tissue (n = 4 hens per treatment). Relative mRNA expression of TNF-α, IL-1β, IL-6, IL-8, and IL-10 was quantified by qPCR in the vagina, UVJ, and infundibulum. No formal a priori power analysis was performed for the cytokine gene-expression endpoints; therefore, these molecular analyses were considered exploratory, particularly with respect to small or moderate treatment effects.

### 2.5. Semen Collection, Semen Preparation, and Artificial Insemination

Semen was collected once weekly from each of the 30 donor roosters using the dorso-abdominal massage technique. At each collection, individual ejaculates were evaluated immediately for sperm concentration, total motility, and viability. Only ejaculates meeting the predefined acceptance criteria (sperm concentration ≥3 × 10^9^ sperm/mL, total motility ≥80%, and viability ≥90%) were included in the pool. These quality assessments were repeated at every collection throughout the 10-week experimental period. A fresh semen pool was prepared for each weekly insemination session from the accepted ejaculates collected on that occasion. The resulting pool was then divided into equal aliquots for the Control, KAN, and AvBD1 treatments before addition of the respective extenders. Thus, within each weekly insemination session, all three semen-containing treatments originated from the same pooled semen source.

Pooled semen was divided into equal aliquots and diluted with the respective treatment extender at a fixed ratio of 1:3 (*v*/*v*; semen). Diluted semen was maintained at 25 °C, and all semen preparation and artificial insemination procedures were completed within 1 h of semen collection. Artificial insemination was performed once weekly between 15:00 and 16:00 h. Each hen received 0.1 mL of diluted semen preparation, whereas hens in the NaCl group received 0.1 mL of sterile 0.9% NaCl using the same insemination procedure. Because all semen-containing treatments were prepared from the same weekly semen pool and diluted using the same ratio, the Control, KAN, and AvBD1 groups received comparable total sperm concentrations within each weekly insemination session.

Eggs were incubated in a forced-air setter (model ROMLUANG-H8000; Romluang Electronics, Chiang Mai, Thailand) at 37.8 °C and 55–60% relative humidity, with automatic turning every hour at an angle of 45° from the vertical. Fertility was assessed by candling on day 7 of incubation. Eggs showing visible embryonic development were classified as fertile, and fertility was calculated as the percentage of eggs classified as fertile relative to the total number of eggs set. Because fertility was assessed by day-7 candling, eggs without visible embryonic development could include both true fertilization failures and very early embryonic losses; these outcomes could not be distinguished retrospectively.

On day 18, eggs were transferred to a forced-air hatcher (DLD-H4000; Romluang Electronics, Chiang Mai, Thailand) maintained at 36.5 °C and 70–75% relative humidity with automatic misting and water-tray humidification. Hatchability was assessed on day 21 and expressed as the percentage of chicks hatched from the total number of fertile eggs [18]. Personnel performing the reproductive outcome assessments were unaware of treatment assignments.

### 2.6. Tissue Collection and Preservation

At 24 h after the final insemination, four hens were randomly selected from each treatment group and euthanized by manual cervical dislocation for tissue collection. The 24 h sampling interval was selected to evaluate a defined post-insemination transcriptional response and was supported by previous evidence of immune-related gene-expression changes in the hen uterovaginal junction at 24 h after mating [21]. Because tissues were collected at a single time point following repeated weekly inseminations, the resulting gene-expression data represent the terminal post-insemination response under this experimental regimen rather than a complete temporal profile.

The reproductive tract was immediately excised, and the vagina, uterovaginal junction (UVJ), and infundibulum were carefully dissected as described by Kheawkanha et al. [22]. These anatomically distinct regions represent key sites involved in semen deposition and sperm passage, sperm storage, and fertilization, respectively.

The tissues were gently rinsed with sterile 0.9% NaCl to remove residual blood and luminal contents. Each sample was transferred individually into a 1.5 mL microcentrifuge tube containing 96% ethanol and stored at −20 °C for approximately one week before RNA extraction. All tissues were collected and immersed in 96% ethanol within 20 min after euthanasia [23]. Treatment identity was not disclosed to the personnel conducting the qPCR analysis.

### 2.7. RNA Extraction and cDNA Synthesis

Total RNA was extracted from the preserved reproductive tissues using TRIzol reagent (Invitrogen, Carlsbad, CA, USA) according to the manufacturer’s instructions, with minor modifications described by Phudkliang et al. [24]. Tissue samples were homogenized using 3 mm tungsten carbide beads (Qiagen, Hilden, Germany) in a TissueLyser LT (Qiagen) at 50 Hz for 2 min and centrifuged at 12,000× *g* for 5 min at 4 °C. After the initial homogenization, 300 μL of chloroform was added, and the mixture was homogenized again at 30 Hz for 2 min. Following incubation at room temperature (25 °C) for 1 min, samples were centrifuged at 12,000× *g* for 15 min at 4 °C. The aqueous phase (approximately 550 μL) was transferred to a new tube, mixed with 700 μL of isopropanol, and centrifuged to precipitate total RNA. The RNA pellet was washed twice with 75% ethanol, air-dried, and prepared for first-strand cDNA synthesis as described by Qi et al. [25] and Chen et al. [26].

Following extraction, RNA concentration and spectrophotometric purity were assessed using a NanoDrop spectrophotometer [Thermo Fisher Scientific, Waltham, MA, USA]. Mean RNA concentrations were 174.77, 184.56, and 193.07 ng/µL for the vagina, UVJ, and infundibulum, respectively. The corresponding mean A260/A280 ratios were 1.85, 1.86, and 1.85, and the mean A260/A230 ratios were 1.92, 1.92, and 1.92, respectively. These measurements indicated RNA yield and spectrophotometric purity before downstream analysis. RNA integrity was not independently assessed using an RNA integrity number (RIN) or gel-based method.

### 2.8. Quantitative Real-Time PCR (qPCR)

Gene expression analysis was performed by qPCR using a CFX Connect™ Real-Time PCR Detection System (Bio-Rad Laboratories, Hercules, CA, USA). The amplification program consisted of an initial denaturation at 95 °C for 10 min, followed by 40 amplification cycles (95 °C for 30 s, 66 °C for 20 s, 72 °C for 20 s, and 86 °C for 5 s), and a final step at 95 °C for 5 s and 75 °C for 1 min. Reactions were performed in a total volume of 20 μL using SYBR^®^ Green I (Invitrogen, Carlsbad, CA, USA) and reagents from the IMMOLASE™ DNA Polymerase kit (Bioline, UK). Primer sequences for the target and reference genes are presented in Table 1.

β-actin was used as the reference gene for normalization. Its amplification was examined in the experimental samples before analysis and was considered sufficiently consistent for use as an internal control in the present dataset. This assessment was preliminary and did not constitute a formal reference-gene stability analysis involving multiple candidate reference genes.

All qPCR reactions were performed in technical duplicate, and the mean Ct value of the two technical replicates was used for subsequent calculations. Relative gene expression was calculated using the 2^−ΔΔCt^ method. For each biological replicate, ΔCt was calculated as Ct*_target_* − Ct*_β-actin_*, and ΔΔCt was calculated relative to the mean ΔCt of the antimicrobial-free Control group within the corresponding tissue–cytokine combination. Individual 2^−ΔΔCt^ values were used for graphical presentation, whereas statistical analyses were performed using ΔCt values.

Quantifiable and non-quantifiable results were classified based on the original qPCR output under the assay conditions used, without retrospectively applying an additional study-specific Ct cutoff. A target transcript was classified as not detected (ND) when β-actin yielded a quantifiable Ct value, but the target transcript did not. ND indicated non-detection under the assay conditions rather than complete biological absence of the transcript. ND observations were neither assigned arbitrary Ct values nor treated as zero expression. Samples without a quantifiable β-actin Ct value were considered not evaluable for ΔCt-based analysis.

Independent primer amplification efficiencies and additional specificity assessments were not available for reporting. Complete records of no-template and no-reverse-transcription controls, as well as additional positive-control data, were not available for retrospective evaluation. These limitations were considered when interpreting the gene-expression findings.

Raw Ct values and calculated ΔCt values for vaginal IL-6 and IL-1β are provided in Supplementary Table S1. Detection frequencies and Ct summaries, including β-actin Ct summaries for evaluable samples, for all cytokine–tissue–treatment combinations are provided in Supplementary Table S2.

### 2.9. Statistical Analysis

Fertility and hatchability of fertile eggs were analyzed using generalized linear mixed models with a binomial distribution and logit link. Fertility was modeled using the numbers of fertile and infertile eggs, whereas hatchability of fertile eggs was modeled using the numbers of hatched and unhatched fertile eggs. Treatment was included as a fixed effect, and hen was included as a random effect to account for repeated observations from the same experimental unit during the 10-week experimental period. Week was included as a repeated effect. Only the three semen-inseminated groups (Control, KAN, and AvBD1) were included in the reproductive performance analyses. When an overall treatment effect was significant, pairwise treatment contrasts were performed using Tukey’s adjustment, and 95% confidence intervals were calculated for treatment differences.

For cytokine gene expression, technical replicates were averaged before statistical analysis and were not considered independent biological replicates. Statistical comparisons were performed using ΔCt values rather than the displayed 2^−ΔΔCt^ values. The vagina, uterovaginal junction, and infundibulum were analyzed separately because each represented a functionally distinct anatomical region. For each cytokine–tissue combination, treatment effects were evaluated using one-way analysis of variance (ANOVA), with treatment as a fixed effect and each sampled hen considered one biological replicate.

Four hens were sampled per treatment. When both β-actin and the target transcript yielded quantifiable Ct values, ΔCt was calculated, and the biological replicate was included in the corresponding analysis. When β-actin was quantifiable, but the target transcript was not, the observation was classified as not detected (ND), and no Ct or ΔCt value was imputed. Samples without a quantifiable β-actin Ct value were considered not evaluable for ΔCt analysis. Thus, the number of quantifiable biological replicates could be fewer than four for a specific cytokine–tissue combination. When the target transcript was not detected in all evaluable NaCl samples, the NaCl group was reported descriptively as ND and excluded from the corresponding quantitative ΔCt-based comparison. Neither ND nor non-evaluable observations were treated as zero expression.

Model assumptions for gene-expression analyses were evaluated using residual diagnostic plots, the Shapiro–Wilk test for normality, and Levene’s test for homogeneity of variances. For analyses in which residual normality was not supported, a Kruskal–Wallis sensitivity analysis was additionally conducted to assess the robustness of the statistical interpretation. When the overall ANOVA treatment effect was significant, pairwise comparisons were performed using Tukey’s adjustment within the corresponding cytokine–tissue analysis.

No additional multiplicity adjustment was applied across the complete set of cytokine–tissue analyses. Accordingly, these molecular analyses were considered exploratory and were interpreted as treatment comparisons within individual tissues rather than as evidence that treatment responses differed among reproductive regions.

Statistical analyses were conducted using SAS software (Version 9.0, SAS Institute Inc., Cary, NC, USA). Statistical significance was declared at *p* < 0.05, and exact *p*-values are reported where appropriate. Reproductive outcomes are presented as model-estimated means ± SEM, whereas gene-expression data are presented as mean ± SEM for graphical presentation.

## 3. Results

### 3.1. Characteristics of Fresh Semen Before Treatment Allocation

Descriptive characteristics of the individual fresh rooster ejaculates that met the predefined semen-quality criteria before pooling and treatment allocation are presented in Table 2.

### 3.2. Effect of Antimicrobial Supplementation on Fertility and Hatchability

Table 3 presents reproductive performance during the 10-week artificial insemination period. Fertility differed significantly among treatments (*p* = 0.0003). Fertility was higher in the KAN and AvBD1 groups than in the antimicrobial-free Control, whereas no significant difference was detected between KAN and AvBD1. Hatchability of fertile eggs did not differ significantly among treatments (*p* = 0.2247).

Pairwise treatment contrasts showed that fertility was 4.96 percentage points higher with KAN than with the Control (95% CI: 1.17 to 8.75; *p* = 0.0098) and 7.54 percentage points higher with AvBD1 than with the Control (95% CI: 3.75 to 11.33; *p* = 0.0002). The estimated difference between AvBD1 and KAN was 2.58 percentage points (95% CI: −1.21 to 6.38; *p* = 0.2182).

For hatchability of fertile eggs, the estimated differences were 2.56 percentage points for KAN versus Control (95% CI: −1.19 to 6.30; *p* = 0.2168), 0.70 percentage points for AvBD1 versus Control (95% CI: −3.04 to 4.44; *p* = 0.8829), and −1.86 percentage points for AvBD1 versus KAN (95% CI: −5.60 to 1.89; *p* = 0.4314). All confidence intervals included zero.

### 3.3. Effect of Antimicrobial Supplementation on Cytokine Gene Expression in the Hen Reproductive Tract

**Figure 2:**
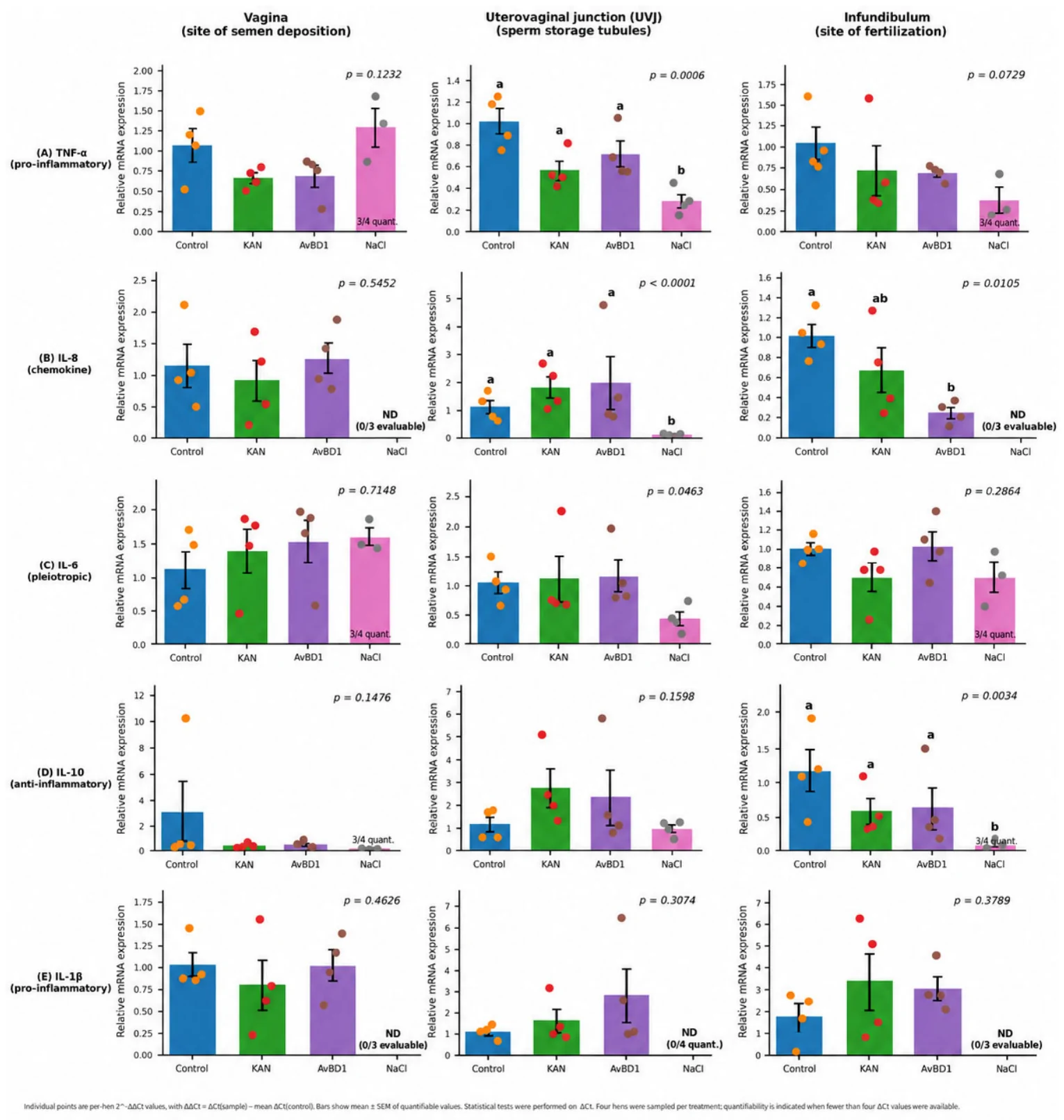
Relative mRNA expression of TNF-α, IL-8, IL-6, IL-10, and IL-1β in the vagina, uterovaginal junction (UVJ), and infundibulum at 24 h after the final insemination. Treatments were Control (antimicrobial-free extender), KAN (kanamycin-supplemented extender), AvBD1 (avian β-defensin 1-supplemented extender), and NaCl (0.9% NaCl sham insemination). Four hens were sampled per treatment. Individual points represent biological replicates with quantifiable ΔCt values, and bars represent mean ± SEM of 2^−ΔΔCt^ values. Statistical analyses were performed using ΔCt values. Different lowercase letters indicate significant Tukey-adjusted pairwise differences (*p* < 0.05). No grouping letters are shown for UVJ IL-6 because the overall treatment effect was significant, but no Tukey-adjusted pairwise comparison reached significance. ND indicates target non-detection in samples with quantifiable β-actin; samples without quantifiable β-actin were considered not evaluable.

Figure 2 presents relative mRNA expression of TNF-α, IL-1β, IL-6, IL-8, and IL-10 in the vagina, uterovaginal junction (UVJ), and infundibulum at 24 h after the final insemination. Treatment effects were evaluated separately within each reproductive region. Detection frequencies and Ct summaries are provided in Supplementary Table S2.

No significant treatment effects were detected for any of the five cytokines examined in the vagina (*p* > 0.05).

In the UVJ, significant overall treatment effects were detected for TNF-α (*p* = 0.0006), IL-8 (*p* < 0.0001), and IL-6 (*p* = 0.0463). For TNF-α and IL-8, expression was lower in the NaCl group than in the three semen-inseminated groups, whereas Control, KAN, and AvBD1 did not differ significantly from one another. Although the overall treatment effect for IL-6 was significant, no Tukey-adjusted pairwise comparison reached significance. IL-10 and IL-1β were not significantly affected by treatment.

In the infundibulum, significant treatment effects were detected for IL-8 (*p* = 0.0105) and IL-10 (*p* = 0.0034). IL-8 expression was lower in the AvBD1 group than in the antimicrobial-free Control, whereas KAN showed an intermediate response. IL-10 expression was lower in the NaCl group than in the three semen-inseminated groups, whereas Control, KAN, and AvBD1 did not differ significantly from one another. No significant treatment effects were detected for TNF-α, IL-6, or IL-1β.

## 4. Discussion

Successful artificial insemination depends not only on the functional competence of sperm but also on the environment encountered within the female reproductive tract. In this study, fertility was higher when rooster semen extenders were supplemented with either kanamycin or AvBD1 than when the antimicrobial-free extender was used, whereas hatchability of fertile eggs did not differ significantly among treatments. Cytokine expression also varied among treatments in selected reproductive regions, although these molecular findings were less consistent than the fertility response. Because bacterial load, sperm transport, sperm persistence within the sperm storage tubules, and early embryonic development were not directly assessed, the mechanisms linking these observations remain uncertain.

The hen reproductive tract provides distinct environments for sperm deposition, storage, and fertilization. Of particular importance is the uterovaginal junction (UVJ), where sperm storage tubules maintain viable sperm after mating or artificial insemination [8,10]. Previous transcriptomic studies show that insemination triggers coordinated epithelial, metabolic, and immune responses within the UVJ and sperm storage tubules [11,12]. Examining cytokine expression in the vagina, UVJ, and infundibulum therefore provides a useful view of the local reproductive environment sperm encounter as they move through the tract. However, because the present study did not test a treatment-by-tissue interaction, the results should be interpreted within each reproductive region rather than as evidence that one region was more responsive than another.

### 4.1. Improved Fertility Following Antimicrobial Supplementation

The main reproductive finding was the higher fertility observed after supplementation with either kanamycin or AvBD1. Bacterial contamination of poultry semen can adversely affect sperm quality and fertilizing capacity, providing a biological basis for using antimicrobial agents in semen extenders [1,2,3]. Nevertheless, this experiment cannot determine whether microbial control drove the fertility response. The semen was used within 1 h of collection, and bacterial load was not measured at the time of insemination. The observed benefit could therefore have resulted from several factors, including better maintenance of sperm function during semen preparation, differences in microbial exposure, interactions with the female reproductive tract, or a combination of these mechanisms.

Interpretation of the fertility endpoint also requires some caution. Fertility was determined by candling on day 7 of incubation, so eggs without visible embryonic development could represent either true fertilization failure or very early embryonic loss. Thus, although fertility differed among treatments, the present data cannot establish whether the treatment effect occurred specifically at fertilization or during the earliest stages of embryo development.

The magnitude of the response was biologically meaningful within the experimental setting. Relative to the antimicrobial-free Control, model-estimated fertility was higher by 4.96 percentage points with KAN (Tukey-adjusted 95% CI: 1.17 to 8.75) and by 7.54 percentage points with AvBD1 (95% CI: 3.75 to 11.33). These differences support a genuine treatment effect under the conditions tested. Whether improvements of this magnitude would translate into a practical advantage under commercial production remains uncertain, however, because economic returns, operational feasibility, and reproductive performance in larger flocks were not evaluated.

Hatchability of fertile eggs showed a different pattern. No statistically significant treatment effect was detected, and the estimated pairwise differences ranged from −1.86 to 2.56 percentage points, with all 95% confidence intervals including zero. This indicates that no clear difference in hatching success among fertile eggs was detected under the conditions tested. Importantly, it does not demonstrate that the treatments were equivalent, because the experiment was not designed or powered as an equivalence or non-inferiority trial.

Our previous chilled-storage study showed antimicrobial and sperm-preserving effects of AvBD1 under different experimental conditions [7], providing the rationale for evaluating AvBD1 here. Those earlier findings, however, should not be used to infer the mechanism operating in the present experiment. Similarly, the higher fertility and the cytokine-expression patterns observed in the current study should be considered separate outcomes. No hen-level association between cytokine expression and fertility was tested; therefore, the transcriptional responses cannot be interpreted as mediators of the fertility effect.

### 4.2. Region-Specific Post-Insemination Cytokine Expression

The cytokine results were more complex than the fertility findings. In the vagina, none of the five cytokines differed significantly among treatments. This suggests that, at the 24 h sampling point, neither kanamycin nor AvBD1 produced a detectable change in vaginal cytokine transcription relative to the antimicrobial-free Control. Post-insemination immune responses can be transient, however, and previous studies have shown that local reproductive responses vary with time after semen exposure [11,12,13]. An absence of differences at 24 h therefore does not exclude responses occurring earlier or later.

A different pattern was observed in the UVJ. Overall treatment effects were detected for TNF-α, IL-8, and IL-6, but examination of the pairwise comparisons showed that the TNF-α and IL-8 differences were largely driven by lower expression in the NaCl sham group. Control, KAN, and AvBD1 did not differ significantly from one another. Because the NaCl group received neither semen nor extender, these findings are more consistent with a response to the semen-containing formulation than with a specific effect of antimicrobial supplementation. TNF-α, IL-6, and IL-8 are involved in inflammatory, epithelial, and chemotactic responses in avian mucosal tissues [13,14,15,16], but the present results do not identify which component of the inseminate was responsible. IL-6 also showed a significant overall treatment effect, although no Tukey-adjusted pairwise comparison was significant. This isolated result should therefore be interpreted cautiously.

The infundibulum provided the clearest cytokine difference associated specifically with AvBD1. IL-8 expression was lower in the AvBD1 group than in the antimicrobial-free Control, while KAN showed an intermediate response. Although this finding suggests that AvBD1 may influence local IL-8 transcription, reduced IL-8 mRNA alone cannot be taken as evidence of reduced leukocyte recruitment or lower inflammatory activity [14,16]. IL-10 showed a different pattern: expression was lower in the NaCl sham group than in all three semen-inseminated groups, whereas Control, KAN, and AvBD1 did not differ from one another. Given the counter-regulatory role of IL-10 in immune homeostasis [17], this response may be related more broadly to exposure to semen or extender components rather than to antimicrobial supplementation itself.

Taken together, the cytokine results provide relatively limited evidence for direct immunomodulation by kanamycin or AvBD1. Most significant pairwise differences involved the NaCl sham group, with infundibular IL-8 representing the main exception. Moreover, changes in mRNA expression do not necessarily correspond to changes in cytokine protein abundance, inflammatory activity, sperm-storage function, or fertility. IL-1β did not differ significantly among treatments at 24 h, and transcripts classified as not detected should be interpreted as non-detection under the assay conditions rather than biological absence [13,28].

These molecular findings should therefore be considered exploratory. The analysis was based on four hens per treatment at a single post-insemination time point, which limits the ability to characterize temporal responses or detect smaller treatment effects. Future studies using larger biological replication, multiple sampling times, and complementary measurements of cytokine proteins, immune-cell responses, and reproductive tract function will be needed to determine whether the transcriptional differences observed here have functional significance.

### 4.3. Further Evaluation of AvBD1 as an Antibiotic-Sparing Component of Poultry Semen Extenders

The higher fertility observed with AvBD1 supplementation supports its further evaluation as a potential antibiotic-sparing component of poultry semen extenders. Importantly, however, the present study does not show that AvBD1 is equivalent to kanamycin. Although fertility did not differ significantly between the AvBD1 and KAN groups, the experiment was not designed as an equivalence or non-inferiority study. The findings therefore indicate that AvBD1 performed favorably under the conditions tested, rather than demonstrating that it can directly replace kanamycin.

This distinction is particularly important because the present experiment did not directly measure antimicrobial activity. Our previous chilled-storage study demonstrated that AvBD1 reduced viable bacterial load while helping to preserve rooster sperm quality and fertility [7], providing the rationale for its use here. Nevertheless, those findings were obtained under different experimental conditions and cannot establish that bacterial suppression was responsible for the fertility response in the current study. Similarly, the cytokine-expression patterns observed here do not demonstrate a direct anti-inflammatory or immunomodulatory action of AvBD1.

Several questions therefore remain before practical application can be considered. The current study did not quantify bacterial contamination at insemination, assess how long AvBD1 remained active after dilution or insemination, evaluate dose–response relationships, or examine the potential for microbial adaptation or resistance selection. Antimicrobial peptides are attractive alternatives to conventional antibiotics because of their broad biological activity, but their practical use also depends on peptide stability, retained antimicrobial activity, safety, and production cost [4,29,30].

The experimental design also did not allow the effects of semen, extender components, and AvBD1 itself to be fully separated. The NaCl group served as a procedural sham but was not an extender-matched control. Future studies could therefore include an extender-only group and, where biologically appropriate, an extender supplemented with AvBD1 without semen to help distinguish the contribution of the inseminate components to reproductive tract responses.

Taken together, these findings justify further investigation of AvBD1 but do not support immediate replacement of conventional antibiotics. Future work should combine direct bacterial quantification and antimicrobial susceptibility testing with peptide-stability studies, dose–response and reproductive-safety assessments, and larger fertility trials under practical production conditions. Evaluation of production cost and operational feasibility will also be necessary before routine application can be considered.

## 5. Conclusions

Supplementation of rooster semen extenders with either kanamycin or AvBD1 was associated with higher fertility than the antimicrobial-free Control, whereas no statistically significant difference was detected in hatchability of fertile eggs. Treatment-associated differences in cytokine mRNA expression were observed in selected regions of the hen reproductive tract, although most significant pairwise differences involved the NaCl sham group rather than differences among the semen-inseminated treatments. Infundibular IL-8 was lower with AvBD1 than with the antimicrobial-free Control, representing the clearest cytokine response specifically associated with AvBD1.

These molecular findings should be interpreted cautiously because cytokine expression was evaluated in only four hens per treatment at a single post-insemination time point and does not establish functional immune effects or a causal relationship with fertility. Likewise, the lack of a significant difference between AvBD1 and kanamycin does not demonstrate treatment equivalence. Overall, the findings support further evaluation of AvBD1 as a potential antibiotic-sparing component of poultry semen extenders, with additional studies needed to confirm its antimicrobial efficacy, reproductive safety, mechanism of action, and practical value under larger-scale production conditions.

## Figures and Tables

**Table 1:** Primer sequences used for quantitative real-time PCR analysis of target and reference genes.

Gene	Primer Sequence (5′→3′)	Reference
β-actin	F: ACCTGAGCGCAAGTACTCTGTCT R: CATCGTACTCCTGCTTGCTGAT	Lin et al. [27]
IL-1β	F: GGGCATCAAGGGCTACAA R: CTGTCCAGGCGGTAGAAGAT	Das et al. [5]
TNF-α	F: CCTGCTGGGGGAATGCTAGG R: AGCGTTGTCTGCTCTGTAGC	Rodríguez et al. [16]
IL-6	F: AGAAATCCCTCCTCGCCAAT R: AAATAGCGAACGGCCCTCA	Elhamouly et al. [14]
IL-8	F: CCGATGCCAGTGCATAGAGA R: GGTGTCTGCCTTGTCCAGAA	Rodríguez et al. [16]
IL-10	F: CAGACCAGCACCAGTCATCAG R: ATCCCGTTCTCATCCATCTTCTCG	Rodríguez et al. [16]

Abbreviations: F, forward primer; R, reverse primer.

**Table 2:** Characteristics of individual fresh rooster ejaculates before pooling and treatment allocation.

Semen Characteristic	Mean ± SD	Range
Semen volume (mL)	0.48 ± 0.04	0.44–0.57
Sperm concentration (×10^9^ sperm/mL)	4.94 ± 0.32	4.25–5.33
Total motility (%)	95.87 ± 0.76	94.25–97.00
Sperm viability (%)	95.81 ± 1.78	93.00–98.23

Values represent individual ejaculates that met the predefined semen-quality criteria before pooling (n = 274 ejaculates collected over 10 weekly collection periods). SD, standard deviation.

**Table 3:** Fertility and hatchability of hens following weekly artificial insemination with antimicrobial-supplemented rooster semen for 10 weeks.

Reproductive Outcome	Control	KAN	AvBD1	*p*-Value
Total eggs set (n)	364	369	355	—
Fertile eggs (n)	236	257	256	—
Fertility (%)	65.27 ± 3.13 ^b^	70.23 ± 3.22 ^a^	72.81 ± 3.21 ^a^	0.0003
Chicks hatched (n)	218	244	239	—
Hatchability of fertile eggs (%)	92.73 ± 1.42	95.29 ± 1.25	93.44 ± 0.75	0.2247

Values for fertility and hatchability of fertile eggs are presented as model-estimated means ± SEM from the binomial generalized linear mixed models described in Section 2.9. Total eggs set, fertile eggs, and chicks hatched are cumulative observed counts over the 10-week experimental period; therefore, model-estimated percentages may differ from crude proportions calculated directly from these counts. Control = semen diluted with IGGKPh extender without antimicrobial supplementation; KAN = semen diluted with IGGKPh extender supplemented with kanamycin at 31.2 µg/mL; AvBD1 = semen diluted with IGGKPh extender supplemented with avian β-defensin 1 at 7.5 µg/mL. Within a row, means with different superscript letters differ significantly based on Tukey-adjusted pairwise comparisons (*p* < 0.05).

## Data Availability

The data are available upon request from the corresponding author.

## Supplementary Materials

The following supporting information can be downloaded at: https://www.mdpi.com/article/10.3390/ani16213525/s1, Table S1: Raw Ct values and calculated ΔCt values for vaginal IL-6 and IL-1β expression; Table S2: Detection frequencies and Ct summaries, including β-actin Ct summaries for evaluable samples, for all cytokine–tissue–treatment combinations.
